# L-Citrulline Improves Recovery of Enterocytes While Not Affecting Gut Microbiota in an In Vitro Model of Chemotherapy-Induced Gastrointestinal Mucositis

**DOI:** 10.3390/biomedicines13092244

**Published:** 2025-09-11

**Authors:** Wally van der Laan, Pablo A. Gallardo Molina, Debby P. Y. Koonen, Hermie J. M. Harmsen, Wim J. E. Tissing

**Affiliations:** 1 Department of Pediatrics, University Medical Center Groningen, Hanzeplein 1, 9713 GZ Groningen, The Netherlands; w.van.der.laan@umcg.nl (W.v.d.L.); d.p.y.koonen@umcg.nl (D.P.Y.K.); 2 Groningen Institute for Evolutionary Life Sciences, University of Groningen, Nijenborgh 7, 9747 AG Groningen, The Netherlands; p.a.gallardo.molina@rug.nl; 3 Department of Medical Microbiology and Infection Prevention, University Medical Center Groningen, Hanzeplein 1, 9713 GZ Groningen, The Netherlands; 4 Prinses Máxima Center for Pediatric Oncology, Heidelberglaan 25, 3584 CS Utrecht, The Netherlands

**Keywords:** L-citrulline, gastrointestinal mucositis, microbiome, methotrexate, melphalan, supportive care, chemotherapy toxicity

## Abstract

**Background/Objectives:** Despite significant advancements in cancer treatment outcomes, side effects, such as gastrointestinal mucositis (GIM), continue to impair patients’ quality of life. The recent literature suggests L-citrulline as a novel treatment for GIM. However, no in vitro model to study the potential for L-citrulline as a treatment for GIM is currently available, and the effect of L-citrulline on the gut microbiota remains unclear. This study aims to propose a suitable in vitro model to study the effect of L-citrulline on the gut microbiota and to determine whether it can mitigate GIM. **Methods:** The CaCo-2 and T84 cell lines were cultured using cell impedance assays and treated with different doses of methotrexate and melphalan to select an appropriate model for L-citrulline research. The selected model was further used to investigate the impact of L-citrulline on gut microbiota cultured using microbial culture assays containing YCFAG. **Results:** Neither CaCo-2 nor T84 cells treated with methotrexate were suitable models for our study due to varying responses to treatment. T84 cells treated with 100 μg/mL melphalan demonstrated a consistent response, making them a suitable model for in vitro research on treatments for GIM. The use of L-citrulline demonstrated potential protective effects, as melphalan-treated enterocytes showed less cellular damage in its presence and slightly reduced enteroaggregative *E. coli* growth. **Conclusions:** L-Citrulline supplementation reduced epithelial cell injury due to melphalan, suggesting therapeutic potential. Further testing is required to determine its efficacy in vivo and clarify the mechanisms underlying this potential benefit.

## 1. Introduction

Childhood cancer is a rare but serious diagnosis with an annual incidence of almost 600 new cases per year in The Netherlands [1]. Despite the overall improvement in survival rates, as a result of advances in tumor classification, novel treatments, and supportive care, the burden of treatment-related complications remains important [2]. One such complication is gastrointestinal mucositis (GIM), which can significantly impact quality of life in pediatric cancer patients [3].

GIM is a self-limiting inflammatory state of the bowel clinically characterized by nausea, vomiting, weight loss, abdominal pain, anorexia, nutritional disorders, and increased susceptibility to gastrointestinal and blood-borne infections [3]. A significant proportion of patients receiving cancer treatment develops GIM throughout their treatment, underlining the urgent need for effective preventive and therapeutic interventions [4].

Chemotherapeutic agents such as methotrexate and melphalan are well-known inducers of GIM. Methotrexate, a folic acid analog, disrupts DNA synthesis by inhibiting the dihydropholate reductase (DHFR) enzyme [5,6]. Melphalan, an alkylating agent, causes DNA damage leading to apoptosis [7]. Despite the numerous attempts to treat or prevent GIM, previous studies have yielded limited success to date [3,8,9,10,11,12,13,14,15,16]. Consequently, research into novel strategies for the prevention and treatment of GIM are highly necessary [3,17].

**Figure 1:**
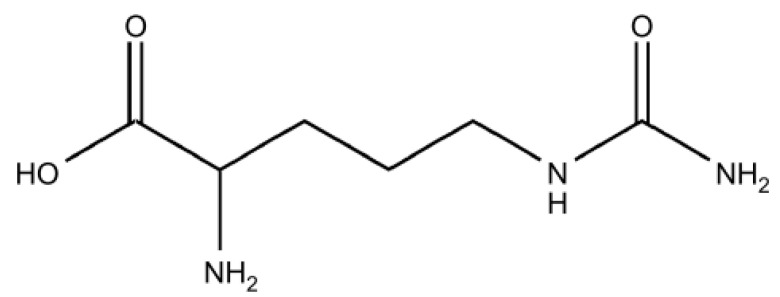
Chemical structure of L-citrulline [18].

One promising candidate is L-citrulline, a non-essential alpha-amino acid produced by the gastrointestinal epithelium [18]. The chemical structure of L-citrulline can be found in Figure 1. The gastrointestinal epithelium is responsible for about 90% of the total bodily L-citrulline produced [18]. Serum levels of L-citrulline have been shown to correlate with the severity of mucosal injury and symptoms in both animal and human studies [19,20,21]. During GIM, the destruction of epithelial cell significantly reduces endogenous L-citrulline synthesis, temporarily turning L-citrulline from a non-essential to a nutritionally essential amino acid [21,22,23]. As a result, L-citrulline has been argued to be the most suitable candidate to investigate as a new therapeutic option to manage GIM [23].

L-citrulline plays a crucial role in gut homeostasis, including nitrous oxide (NO) production, which is thought to mitigate chemotherapy-induced oxidative stress by reducing reactive oxygen species (ROS) [23,24,25]. A deficiency of L-citrulline may, therefore, lead to an exacerbation of gut epithelial injury [23]. This leads to two primary hypotheses: (1) that L-citrulline becomes a nutritionally or metabolically limiting factor for gastrointestinal epithelial cell repair during GIM, and (2) that L-citrulline may directly reduce mucosal inflammation, therefore mitigating injury severity. Additionally, L-citrulline may interact with gut microbiota to reduce local inflammation and prevent or treat GIM. Dietary supplementation of L-citrulline could, therefore, represent a simple, biologically plausible strategy to reduce the incidence or severity of GIM in patients receiving cancer treatment.

However, before moving to in vivo trials, in vitro testing of L-citrulline is required to explore its potential therapeutic use in a controlled environment. Additionally, it is essential to consider the broader impact of L-citrulline particularly on the gut microbiota, which has a known bidirectional association with GIM [26,27,28,29,30]. Changes in intraluminal L-citrulline levels may change gut microbiota composition, adding or resting from its protective effects. Thus, understanding the interactions because of L-citrulline and gut microbiota is crucial for evaluating its therapeutic viability for GIM.

This study has three primary aims: (1) to develop an in vitro cell culture model of chemotherapy-induced GIM, using methotrexate and melphalan; (2) to investigate the effect of L-citrulline supplementation on enterocyte growth and recovery after methotrexate- and melphalan-induced cell injury; and (3) to evaluate the impact of L-citrulline supplementation on the growth of representative gut microbiota species.

## 2. Materials and Methods

### 2.1. Cell Cultures

Human colorectal epithelial CaCo-2 (ATCC HTB-37™, passages 27–37) and T84 (ATCC CCL-248™, passages 8–18) cells were cultured at 37 °C in a humidified atmosphere with 5% CO_2_ [31]. CaCo-2 cells were maintained in high-glucose DMEM with GlutaMAX™ (Gibco™, Thermo Fisher Scientific, Waltham, MA, USA), supplemented with 10% heat-inactivated fetal calf serum (FCS; Gibco™), 1% Antibiotic-Antimycotic (Gibco™, Thermo Fisher Scientific, Waltham, MA, USA), and 1% essential amino acids (EAA; Gibco™). Notably, the EAA supplement did not contain L-citrulline. T84 cells were cultured in DMEM/F-12 with GlutaMAX™ (Gibco™, Thermo Fisher Scientific, Waltham, MA, USA), supplemented with 10% heat-inactivated FCS and 1% Antibiotic-Antimycotic.

Cells were passaged at approximately 80% confluency using 0.25% trypsin-EDTA with phenol red (Gibco™, Thermo Fisher Scientific, Waltham, MA, USA). Detached cells were centrifuged and resuspended in fresh complete medium. Cell numbers were assessed using trypan blue exclusion (Gibco™, Thermo Fisher Scientific, Waltham, MA, USA) and counted in duplicate with an automated cell counter (LUNA-II™, Logos Biosystems, Villeneuve d’Ascq, France). All cultures were routinely tested for contamination and only used within the defined passage range.

### 2.2. Bacterial Cultures

The bacterial strains used in this study included *Bacteroides fragilis* (HTF-786 (N8HP−)), *Bifidobacterium longum* sub. *longum* (HTF-845 IN4JP + J), *Akkermansia muciniphila* (K9), *Megamonas funiformis* (HTF-872 N3KP−), *Bifidobacterium bifidum* (HTF-772 (N9HP+)), *Roseburia intestinalis* (1-82-3), *Anaerobutyricum hallii* (L2-7 colony 2), *Faecalibacterium prausnitzii* (A2-165 and 238), and enteroaggregative *Escherichia coli*. The strains were identified using Gram staining and MALDI-TOF MS prior to use in experiments [32,33]. All strains were cultured in Yeast, Casitone, Fatty Acid and Glucose medium (YCFAG), details of formulation are found in Table A1 (Appendix A). YCFAG was autoclaved at 121 °C, supplemented with vitamins, and filtered before its use in bacterial culturing or experiments. Bacterial cultures were kept at 37 °C in a BACTRON anaerobic chamber (Sheldon Manufacturing Inc., Cornelius, ON, USA). Oxygen levels in the anaerobic chamber were kept below 0.1% at all times.

### 2.3. Chemical Compounds

Methotrexate (Teva Nederland, Haarlem, The Netherlands) was obtained from the in-house pharmacy of the University Medical Center Groningen as a 1 mg/mL solution and stored at 4 °C for a maximum of 7 days in light-protected conditions. Working dilutions (100, 300, 600, 1000, 2000, and 10,000 ng/mL) were prepared in complete growth medium on the day of the experiment. Melphalan (Sun Pharmaceutical Industries, Hoofddorp, The Netherlands) was reconstituted to 5 mg/mL in the manufacturer-provided diluent immediately prior to use, and final concentrations (50, 80, 100, and 150 μg/mL) were freshly prepared in complete growth medium. The concentrations used for both methotrexate and melphalan, in the L-citrulline experiments, reflect the serum levels reported in patients [34,35].

L-citrulline (≥98% purity (TLC), non -sterile., TCI Chemicals™, Tokyo, Japan) was prepared fresh in either complete cell culture medium (1, 3, 10, 30, and 100 mM) or YCFAG (0.1, 1, and 10 mM) for cell experiments or bacterial cultures, respectively. All L-citrulline dilutions were sterile-filtered using 0.2 µm filters immediately before use. While L-citrulline concentrations are supraphysiological, they fall within the range of doses used in dietary supplementation studies [36,37].

All solutions were equilibrated to room temperature to prevent thermal shock and warmed just before experiments, for only for 2 min to 37 °C in a water bath, to minimize degradation.

### 2.4. xCELLigence Real-Time Cell Analysis

To establish an in vitro model of GIM, the optimal seeding density for CaCo-2 and T84 cells was determined using XCELLigence real-time cell analysis (RTCA; Agilent™, Santa Clara, CA, USA) [38,39]. Cells were seeded in 16-well E-plates and monitored for 24 h to identify the density that yielded exponential growth, as measured by the Cell Index (CI), a unitless parameter reflecting impedance across gold microelectrodes. Background readings were recorded every 5 min for 1 h, prior to seeding, after which cell proliferation was monitored every 15 min at 37 °C and 5% CO_2_.

To simulate GIM, a sublethal dose of chemotherapy was defined as the concentration that induced a maximal decrease in CI followed by recovery to control-like proliferation after compound removal. Chemotherapy (either methotrexate or melphalan) was added at 24 h, and growth medium was fully refreshed at defined time points to mimic clinical clearance of cytotoxic agents. L-citrulline was co-administered with chemotherapy or control medium at 24 h. Its effect on cell recovery was measured by comparing CI values following compound removal at 28 h (melphalan) and 48 h (methotrexate). This difference in time points is because the cytotoxic effect of melphalan is faster than the cytotoxic effect of methotrexate. All experimental conditions were tested in duplicate and each experiment was independently duplicated.

### 2.5. Biotek Synergy HT Microplate Reading

The impact of L-citrulline on bacterial growth was assessed by measuring optical density (OD) over time using a Synergy HT microplate reader (Biotek™, Agilent™, Santa Clara, CA, USA). Exponentially growing cultures of each bacterial strain were inoculated into 96-well plates containing YCFAG supplemented with L-citrulline in the pre-defined concentrations. For each condition, 10 µL of bacterial suspension (OD_600_ = 0.8) was added to 190 µL of medium. Negative controls (medium only) were included in triplicate. OD_600_ was recorded every 30 min over 24 h period with orbital shaking prior to each read. All conditions were tested in technical triplicates.

### 2.6. Statistical Analysis

Statistical analysis was performed using Graphpad Prism version 10.4.2 (GraphPad Software, San Diego, CA, USA). One-way analysis of variance (ANOVA) was used to determine differences between groups. When significant, post hoc comparisons were conducted using Tukey’s Honestly Significant Difference (HSD) test. A *p*-value < 0.05 was considered statistically significant.

## 3. Results

### 3.1. T84 Cells Treated with Melphalan Constituted the Most Suitable Model for Investigating GIM In Vitro

**Figure A1:**
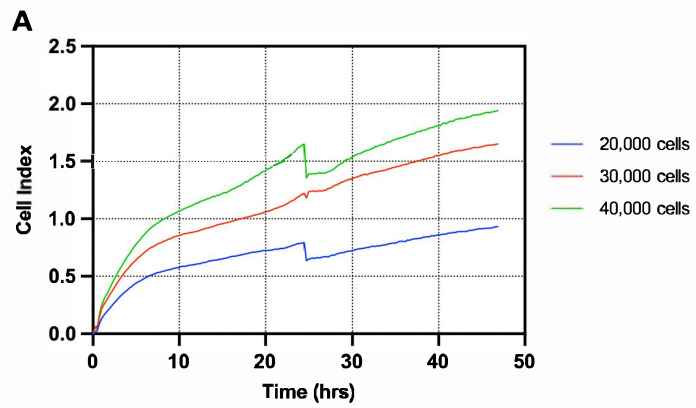

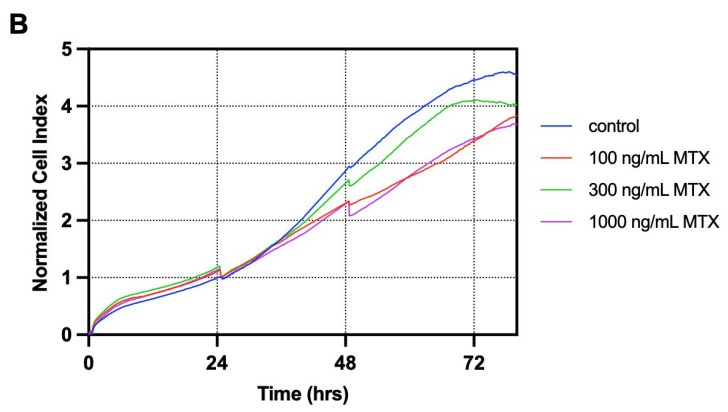
(**A**) Growth curves of CaCo-2 cells cultured at different seeding densities (20,000, 30,000, and 40,000 cells per well). (**B**) Growth curves of CaCo-2 cells treated with different concentrations of methotrexate (MTX) (normalized at *t* = 24 h).

CaCo-2 cells were seeded on an xCELLigence E-plate at densities of 10,000, 20,000, 30,000 and 40,000 cells (Figure A1A (Appendix B)). The cells did not enter an exponential phase at *t* = 24 h, but started growing exponentially after *t* = 24 h in wells seeded with 40,000 cells. Therefore, for the model, a seeding density of 40,000 cells was chosen with the methotrexate dose to be added at *t* = 48 h to ensure treatment occurred during the exponential phase. Subsequent treatment of CaCo-2 cells with 0, 100, 300, 600, 1000, 2000 and 10,000 ng/mL methotrexate revealed no effect of methotrexate on these cells (Figure A1B (Appendix B)). As a result, we concluded that CaCo-2 cells are not susceptible to methotrexate-induced cell death, and switched to a T84 cell-based model treated with methotrexate.

**Figure A2:**
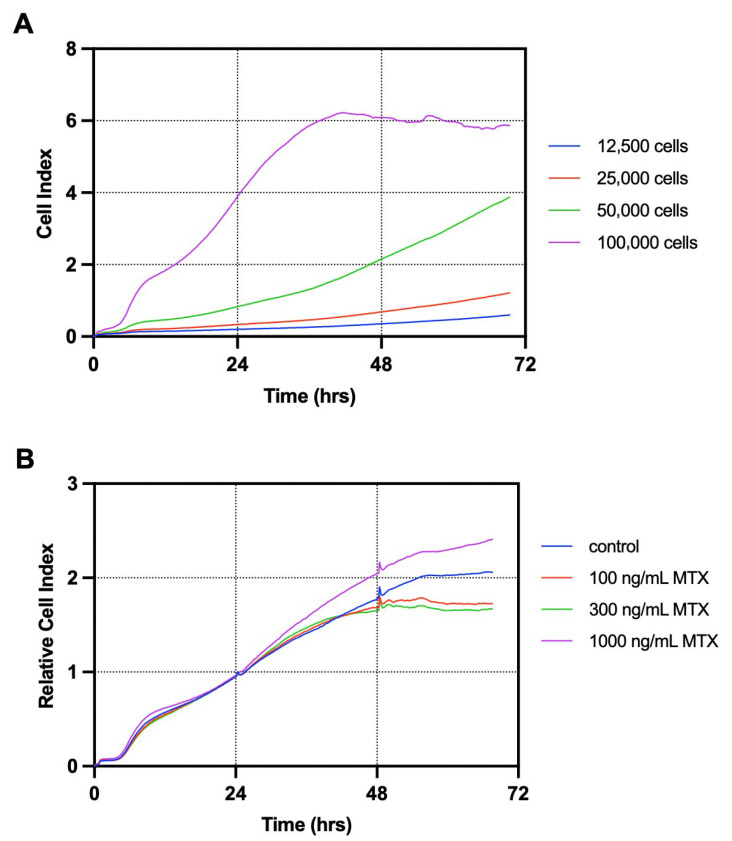
(**A**) Growth curves of T84 cells cultured at different seeding densities (12,500, 25,000, 50,000, and 100,000 cells per well). (**B**) Growth curves of T84 treated with different concentrations of methotrexate (MTX) (normalized at *t* = 24 h).

T84 cells were seeded on an xCELLigence E-plate at densities of 12,500, 25,000, 50,000 and 100,000 cells (Figure A2A (Appendix B)). Wells seeded with 12,500 and 25,000 cells did not grow exponentially during the measurement at all. Wells seeded with 50,000 cells and 100,000 cells did grow exponentially during the measurement, though the effect at *t* = 24 h was greater in wells seeded with 100,000 cells. As a result, a seeding density of 100,000 cells was chosen for the T84 cell-based model. Subsequent treatment of T84 cells with 100, 300, and 1000 ng/mL methotrexate revealed no cytotoxic effect of methotrexate either (Figure A2B (Appendix B)). A subsequent trial with 10,000 ng/mL methotrexate in T84 cells revealed no effect of methotrexate on these cells (Figure A2B Appendix B)). As a result, we concluded that T84 cells are not susceptible to methotrexate-induced cell death, and substituted methotrexate for melphalan.

**Figure A3:**
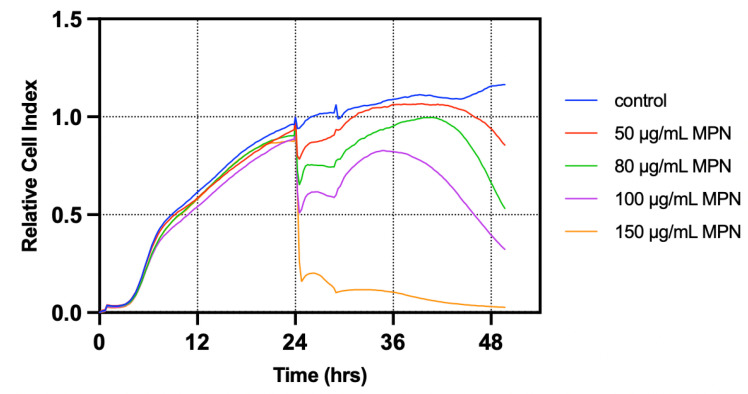
T84 cells are treated with melphalan (MPN) at different doses at *t* = 24 h and melphalan subsequently removed at *t* = 28 h.

To determine the appropriate concentration of melphalan for the model, T84 cells seeded on an xCELLigence E-plate at 100,000 cells per well were treated with 50, 80, 100, and 150 μg/mL single doses of melphalan at *t* = 24 h (Figure A3 (Appendix B)). Cells treated with 50 and 80 μg/mL melphalan did not differ from the control wells, while cells treated with 150 μg/mL melphalan were injured to the extent they did not regrow after removal of melphalan at *t* = 28 h. Resultingly, a dose of 100 μg/mL melphalan was selected for the model.

### 3.2. L-Citrulline Supplementation Improved Cell Recovery in a T84 Cell-Based Model of GIM

**Figure A4:**
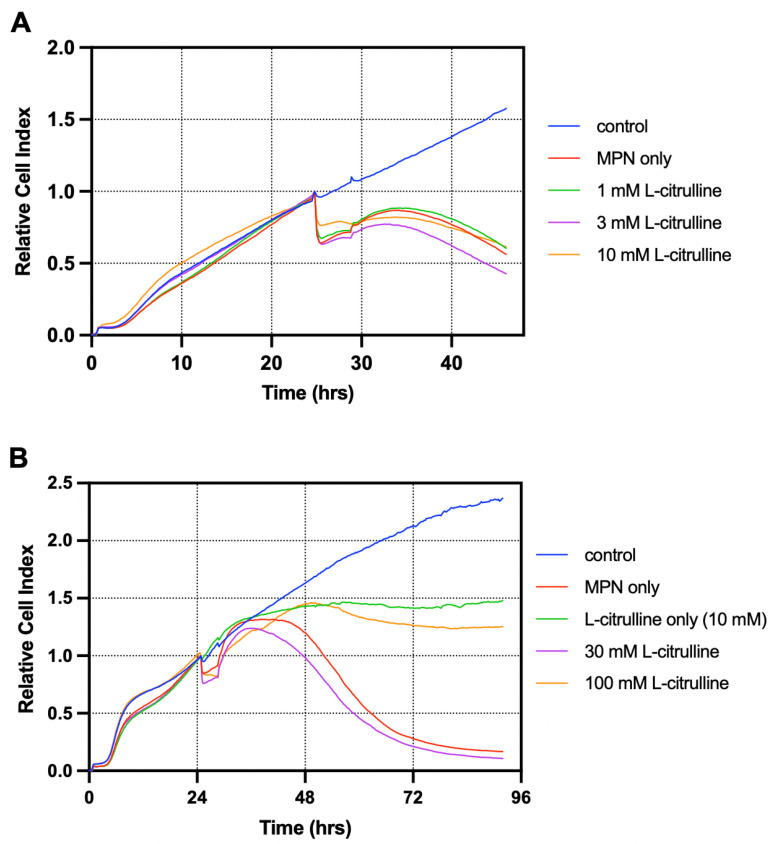
(**A**) T84 cells are treated with melphalan (MPN) and L-citrulline (doses 1 mM, 3 mM, and 10 mM) or control at *t* = 24, and melphalan is subsequently removed at *t* = 28 h. (**B**) T84 cells are treated with melphalan (MPN) and L-citrulline (doses 30 mM and 100 mM) or control at *t* = 24 and melphalan is subsequently removed at *t* = 28 h.

In the selected melphalan-treated T84 cell-based model, we investigated the effect of L-citrulline on the growth curves seeded cells in an xCELLigence E-16 plate. L-citrulline (1, 3, 10, 30, or 100 mM) or a control was added at *t* = 24 h of the experiment. No effect on the growth curves was found between wells treated with L-citrulline at doses of 1, 3 or 10 mM, and cells not treated with L-citrulline (Figure A4A (Appendix B)). A subsequent experiment was carried out investigating whether higher doses of L-citrulline (30 and 100 mM) at *t* = 24 h showed an effect on the growth curves. This revealed that, although no effect was visible in cells treated with 30 mM L-citrulline, cells treated with 100 mM L-citrulline showed significantly more growth compared to the melphalan-treated cells receiving no L-citrulline, also after post hoc analysis (Figure A4B (Appendix B); *p* < 0.0001). Nevertheless, the growth curve of cells treated with 100 mM L-citrulline was statistically significantly lower compared to the control wells after post hoc analysis (*p* < 0.0001).

### 3.3. L-Citrulline Supplementation Did Not Affect the Growth Rate of Selected Gut Bacteria

**Figure A5:**
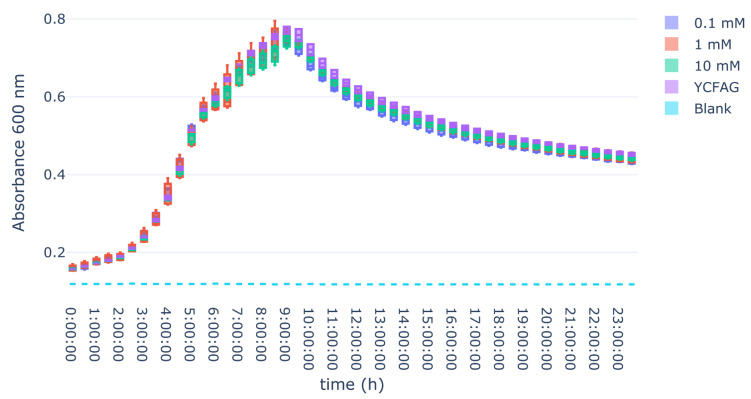
Growth curve of *Roseburia intestinalis* when exposed to L-citrulline or control medium.

**Figure A6:**
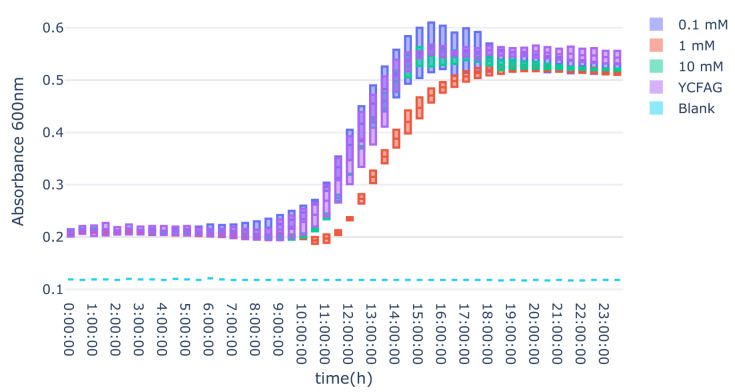
Growth curve of *Megamonas funiformis* when exposed to L-citrulline or control medium.

**Figure A7:**
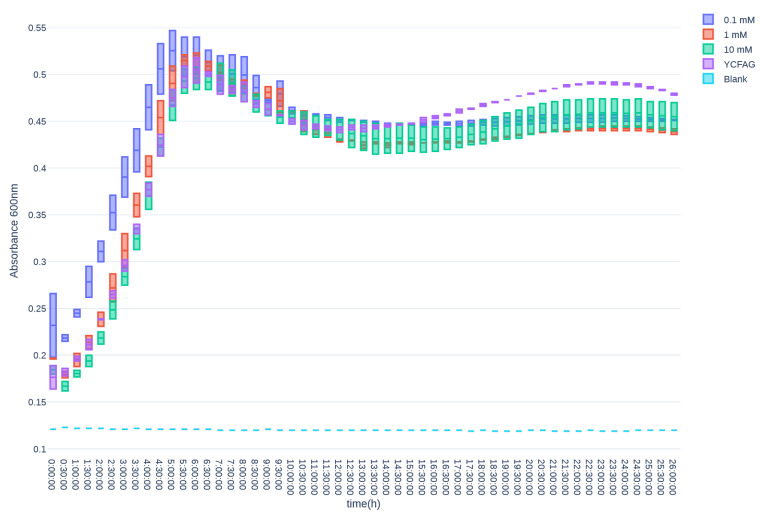
Growth curve of *Faecalibacterium prausnitzii* (A2-165) when exposed to L-citrulline or control medium.

**Figure A8:**
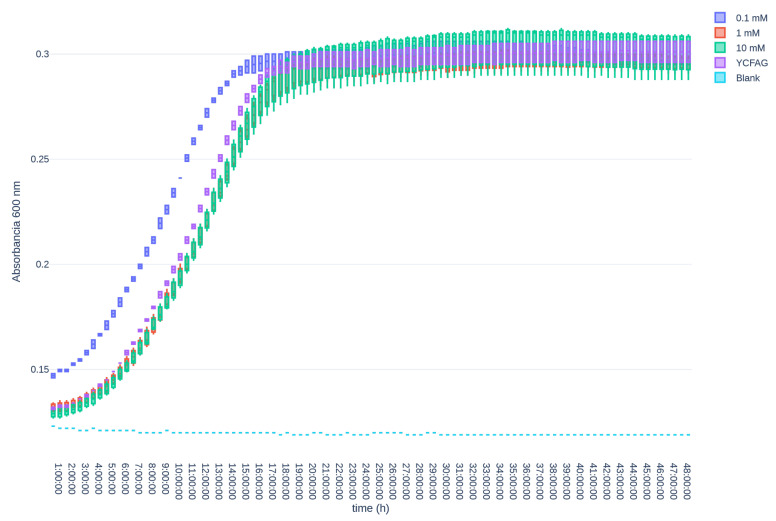
Growth curve of *Faecalibacterium prausnitzii* (HTF238) when exposed to L-citrulline or control medium.

**Figure A9:**
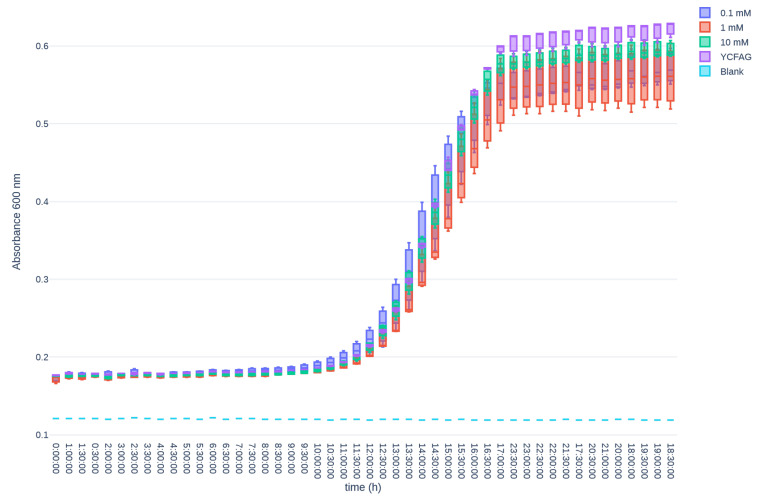
Growth curve of *Bifidobacterium longum* sub. *longum* when exposed to L-citrulline or control medium.

**Figure A10:**
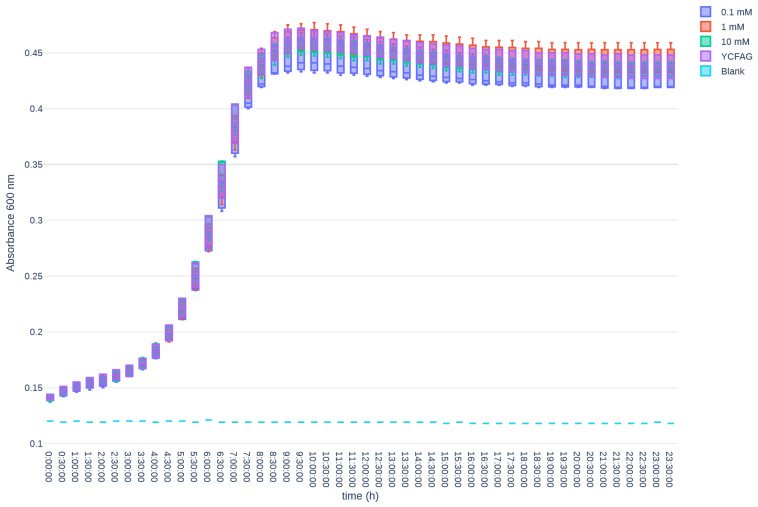
Growth curve of *Bifidobacterium bifidum* when exposed to L-citrulline or control medium.

**Figure A11:**
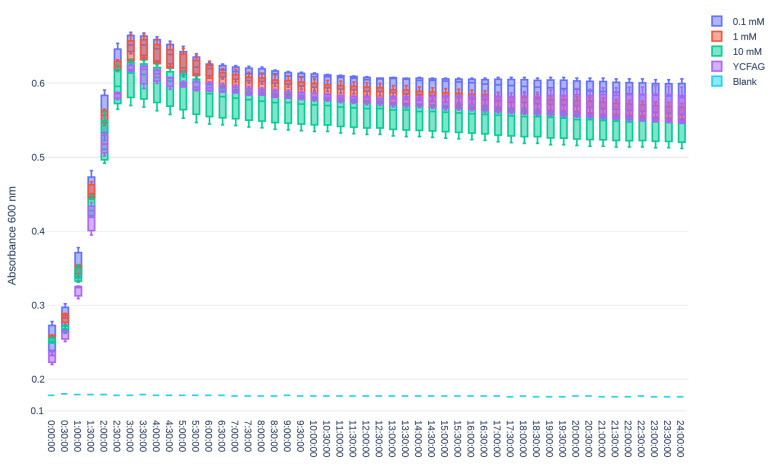
Growth curve of *Bacteroides fragilis* when exposed to L-citrulline or control medium.

**Figure A12:**
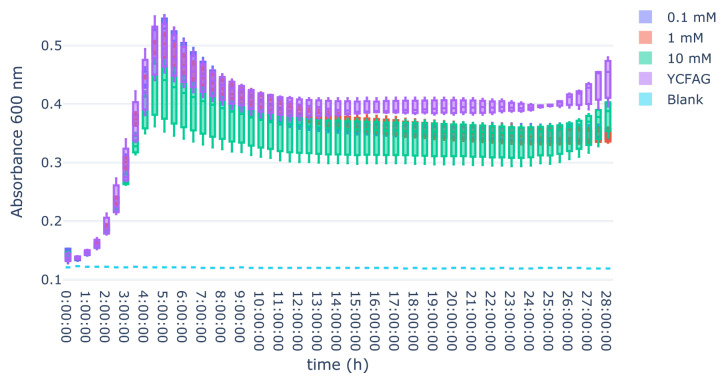
Growth curve of *Anaerobutyricum halli* when exposed to L-citrulline or control medium.

**Figure A13:**
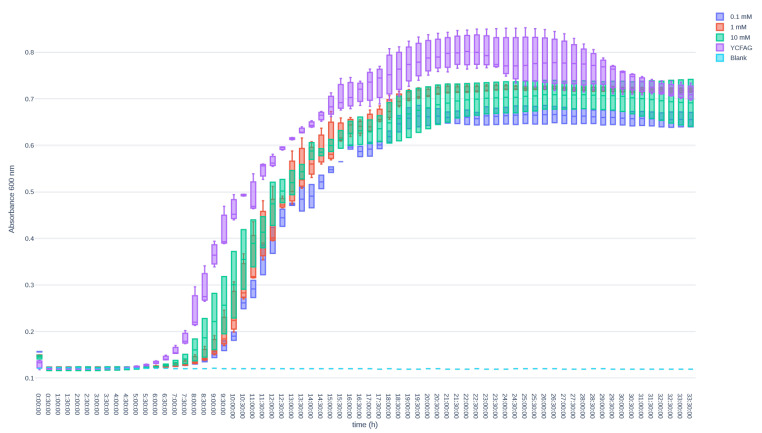
Growth curve of *Akkermansia muciniphila* when exposed to L-citrulline or control medium.

**Figure A14:**
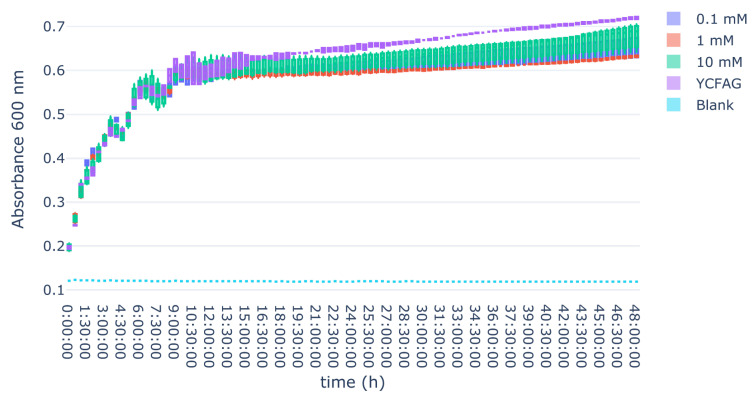
Growth curve of enteroaggregative *Escherichia coli* when exposed to L-citrulline or control medium.

The following gut bacteria in this study showed no significant difference in growth rates when L-citrulline was added to the growth medium: *B. longum* sub. *longum* (*p* = 0.639), *M. funiformis* (*p* = 0.265), *B. bifidum* (*p* = 0.839) *R. intestinalis* (*p* = 0.892), and *F. prausnitzii* (*p* = 0.101). *B. fragilis* showed a significant difference in growth curves through one-way ANOVA (*p* < 0.001), but this disappeared after post hoc analysis. *A. muciniphila* showed a significant difference in growth curves at *t* = 24 h as well (*p* < 0.001) with post hoc analysis revealing this difference was only significant in the comparison of 0.1 mM L-citrulline with only YCFAG (*p* = 0.002). *A. hallii* showed a significant difference in growth curves as well (*p* < 0.001), with post hoc analysis revealing this holds true for each comparison except for 0.1 mM versus 1 mM (*p* = 0.997). *E. coli* showed a significant difference in growth curves (*p* < 0.001) and post hoc analysis showed several comparisons to be statistically significant: only YCFAG versus 0.1 mM (*p* < 0.001), only YCFAG versus 1 mM (*p* < 0.001), only YCFAG versus 10 mM (*p* = 0.004), and 1 mM versus 10 mM (*p* = 0.02). *E. coli* overall grew marginally but significantly worse when the growth medium contained L-citrulline. All complete growth curves are displayed in Figure A5, Figure A6, Figure A7, Figure A8, Figure A9, Figure A10, Figure A11, Figure A12, Figure A13 and Figure A14.

## 4. Discussion

In this study, we developed an in vitro model of melphalan-induced GIM and showed that L-citrulline treatment partially prevented melphalan-induced injury to gastrointestinal cells. Furthermore, we showed that L-citrulline did not have an effect on most of the gut microbiota examined in this study. Altogether this suggests L-citrulline as a potential novel treatment for chemotherapy-induced gastrointestinal mucositis.

This study is not the first to develop an in vitro model of GIM. Other T84-based models exist and have been used to investigate novel therapies for GIM, though none have been performed in an xCELLigence real-time cell analysis model [40,41,42,43]. These other cell-based models primarily measure gut permeability or the production of inflammatory cytokines as proxies for gastrointestinal injury, while the model presented in this article directly measures cell death and growth. Although the existing models perform viability assays, none of these occur in real-time. Therefore, the presented model contributes to existing models by allowing for real-time analysis and observation of growth and death effects overtime. Furthermore, we showed that in our experiments T84 is not susceptible for methotrexate-induced cell death. CaCo-2 cells have also previously been used to develop other chemotherapeutic agent-based models of GIM, particularly irinotecan and 5-fluorouracil (5-FU) [43,44,45,46,47,48,49]. In addition, despite our findings suggesting CaCo-2 cells treated with methotrexate do not form a reliable model of GIM in real-time cell analysis, others did find an effect of methotrexate on CaCo-2 cells [50]. An explanation cannot be sought in the dosing, since the maximum dose of methotrexate used by Youmba and colleagues was lower compared to the maximum dose in this study (10 ng/mL versus 20,000 ng/mL) [50]. Additionally, Sukhotnik and colleagues [51]. The cause for this difference is likely due to the difference in models used. The xCELLigence real-time cell analysis does not measure permeability and, therefore, may not always be able to reproduce the results of studies using other methodologies. Other human cell-line based models of GIM were developed in HT-29, HCT-116, THP-1, and U-937, though none of these models made use of real-time cell analysis either [41,49,52,53,54].

As far as we know, the present study is the first in vitro study in which L-citrulline is studied as a potential intervention to treat or prevent GIM in a model of chemotherapy-induced GIM. A single previous study in a mouse model found that L-citrulline supplementation at a dose of 1 g per kilogram body weight per day had a positive effect on gut permeability when supplemented alongside 5-FU [54]. This study, therefore, aligns with previous findings of the mucoprotective effect of L-citrulline. Different chemotherapeutic agents may present with different inflammatory mechanisms to cause GIM, however, meaning that this effect may be limited to the effect observed in the present study’s melphalan-based model. Whether the effect of L-citrulline is chemotherapeutic agent-specific should, therefore, be investigated in future studies. Animal studies on L-citrulline in conditions negatively affecting the gut mucosa other than chemotherapy-induced GIM also revealed an ameliorating effect of L-citrulline supplementation on the growth of gastrointestinal cells, intestinal perfusion, and local mucosal inflammation [55,56,57,58,59,60,61,62,63,64,65]. The reason behind the effect of L-citrulline on gastrointestinal cells observed in this study may, therefore, be the direct effect that L-citrulline has on the growth of these cells. Future in vivo studies may reveal, however, to what extent the NO cycle and gut microbiota may be involved in the further potential effect of L-citrulline on GIM.

GIM is bilaterally associated with gut microbiota dysbiosis [26,27,28,29,30]. The present study revealed L-citrulline to have little effect on a selection of gut microbiota. Most bacteria included in this study showed no significant difference in growth when exposed to L-citrulline. Although some comparisons in *B. fragilis* and *A. muciniphila* showed a significant difference, post hoc analysis revealed these were not consistent across all conditions tested. This suggests that it is unlikely that there exists a genuine difference beyond statistical significance for these two bacteria. Although the growth of *A. hallii* was significantly affected, this effect is unlikely to be relevant in vivo. Despite the present study not performing mechanistic research to the exact relationship between L-citrulline and these gut bacteria, this is likely due to these bacteria not being metabolically dependent on L-citrulline concentrations.

Results of previous in vivo studies on the effect of L-citrulline on gut microbiota composition have been conflicting, however. One study found supplementation of L-citrulline to bear no effect on the gut microbiota composition of C57BL/6J mice [61]. A different study in rats showed L-citrulline to increase the abundance in particular of Bacteroides species, though an effect of L-citrulline on *B. fragilis* was not found in the presented study [57]. A study performed in poultry found a dose-dependent effect of L-citrulline on the growth of, for example, *Prevotellaceae*, *Blautia*, *Lactobacilli*, and *Campylobacterota* [66]. Finally, in pigs, L-citrulline supplementation was found to positively affect the diversity of their gut microbiota compositions [67]. In contrast, our in vitro data suggest that a possible effect on the microbiome might be an indirect effect, instead of a direct effect on bacterial growth. No previous literature exists on the antibacterial properties of L-citrulline against *E. coli*, though our study suggests there is a potential positive effect of L-citrulline on *E. coli* growth. Previous studies indicate that *E. coli* is likely to become a dominant microbe in patients affected by GIM, resulting in worse treatment outcomes [68,69]. This intervention could, therefore, potentially mitigate GIM by reducing the growth of *E. coli*.

In conclusion, our study suggests an ameliorating effect of L-citrulline on gastrointestinal cells treated with melphalan while not affecting the gut microbiota. Our conclusions warrant further in vivo studies to confirm the effect of L-citrulline on GIM.

## Data Availability

The data that support the findings of this study are not openly available due to reasons of sensitivity and are available from the corresponding author upon request. Data are located in controlled access data storage at the University Medical Center Groningen.

## Appendix A

**Table A1:** YCFAG composition [70]. Concentrations are provided in grams per liter (g/L) unless specified otherwise. Volume units are provided where relevant.

Component	Concentration
Glucose	4.5
Casitone	10
Yeast Extract	2.5
Sodium Bicarbonate	4
Dipotassium Hydrogen Phosphate	0.45
Potassium Dihydrogen Phosphate	0.45
Sodium Chloride	0.9
Magnesium (II) Sulfate Heptahydrate	0.09
Calcium Chloride Dihydrate	0.12
Sodium Acetate	2.7
Cysteine	1
Resazurin (0.02%)	5 mL
Hemin (0.2%)	5 mL
Valerate (≥99% purity)	100 µL
Propionate (≥99% purity)	600 µL
Isobutyrate (≥99% purity)	100 µL
Pink Vitamin Mixture	1 mL
Yellow Vitamin Mixture	1 mL
** Pink Vitamin Mixture **	** Concentration (mg/L) **
Biotin	1
Cobalamin	1
p-Aminobenzoic Acid	3
Folic Acid	5
** Yellow Vitamin Mixture **	** Concentration (mg/L) **
Thiamine	5
Riboflavin	5
